# Dose and type of crystalloid fluid therapy in adult hospitalized patients

**DOI:** 10.1186/2047-0525-2-17

**Published:** 2013-08-06

**Authors:** Annemieke Smorenberg, Can Ince, AB Johan Groeneveld

**Affiliations:** 1 Department of Intensive Care, Erasmus Medical Centre, ‘s-Gravendijkwal 230, 3015, CE, Rotterdam, The Netherlands

**Keywords:** Shock, Hypovolemia, Fluid resuscitation, Normal saline, Ringer’s lactate, Hypertonic saline

## Abstract

**Objective:**

In this narrative review, an overview is given of the pros and cons of various crystalloid fluids used for infusion during initial resuscitation or maintenance phases in adult hospitalized patients. Special emphasis is given on dose, composition of fluids, presence of buffers (in balanced solutions) and electrolytes, according to recent literature. We also review the use of hypertonic solutions.

**Methods:**

We extracted relevant clinical literature in English specifically examining patient-oriented outcomes related to fluid volume and type.

**Results:**

A restrictive fluid therapy prevents complications seen with liberal, large-volume therapy, even though restrictive fluid loading with crystalloids may not demonstrate large hemodynamic effects in surgical or septic patients. Hypertonic solutions may serve the purpose of small volume resuscitation but carry the disadvantage of hypernatremia. Hypotonic solutions are contraindicated in (impending) cerebral edema, whereas hypertonic solutions are probably more helpful in ameliorating than in preventing this condition and improving outcome. Balanced solutions offer a better approach for plasma composition than unbalanced ones, and the evidence for benefits in patient morbidity and mortality is increasing, particularly by helping to prevent acute kidney injury.

**Conclusions:**

Isotonic and hypertonic crystalloid fluids are the fluids of choice for resuscitation from hypovolemia and shock. The evidence that balanced solutions are superior to unbalanced ones is increasing. Hypertonic saline is effective in mannitol-refractory intracranial hypertension, whereas hypotonic solutions are contraindicated in this condition.

## Review

The debate on fluid treatment of the surgical and critically ill patient is ongoing, partly because of insufficient data, historical and geographical differences, and poor training [1-3]. This includes the type and amount of fluids to be given when a patient is considered to be hypovolemic with a low plasma volume, or in shock, with a low or normal plasma volume, and in need of fluids for resuscitation or subsequent maintenance treatment [2,4,5]. Furthermore, the composition, presence or absence of buffer (so-called balanced and unbalanced solutions, respectively) and the additions of certain electrolyte, such as a potassium, calcium and magnesium, are controversial issues.

This review aims to describe the place of various crystalloid fluids in the treatment of adult hospitalized patients, in a narrative manner for educational purposes, leading through various clinical conditions and considerations by evaluation of various studies. We first discuss normal fluid and electrolyte composition of the body, balances and doses of crystalloid fluids, before embarking upon a discussion of relative pros and cons, under various circumstances, of the variously composed crystalloid solutions currently available. We will not discuss any experimental animal-derived nor pediatric data and studies. We end with a discussion of specific electrolyte repletion.

### Fluid and electrolyte balance of the body

Table 1 shows the daily fluid balance of a healthy 70 kg male adult with 45 l of total body water (60% of body weight) [6]. Daily requirements are 80 to 120 mEqsodium, 50 to 100 mEq potassium, 2 to 4 mEq calcium and 20 to 30 mEq magnesium. Fluid and electrolyte therapy aims to correctdeficits, and maintain daily requirements and normal concentrations in body fluids (Table 2).

**Table 1:** Water balance per 24 hour in a healthy 70 kg male adult

**Intake**		**Output**	
Liquids	1,400 ml	Urine	1,500 ml
Water in food	850 ml	Skin	500 ml
Water by oxidation	350 ml	Respiratory tract	400 ml
		Stool	200 ml
Total	2,600 ml	Total	2,600 ml

**Table 2:** Composition of bodily fluids

	**Na**^**+ **^**(mmol/l)**	**K**^**+ **^**(mmol/l)**	**Ca**^**2+ **^**(mmol/l)**	**Mg**^**2+ **^**(mmol/l)**	**HCO**_**3 **_**(mmol/l)**	**Lactate(mmol/l)**	**Other buffer (mmol/l)**	**Cl**^**-**^**(mmol/l)**	**Organic acid (mmol/l)**	**Protein**
**Plasma**	142	4	5	2	101	27	2	1	6	16
**Plasma water**	153	4.3	5.4	2.2	109	29	2.2	1	6.5	17
**Interstitial water**	139	4	5	2	114	31	2	1	7	1
**Intracellular water**	10	160	2	26	3	10	100	20		65

In hospitalized or critically ill patients, however, fluid and electrolyte requirements may be increased following fever, vomiting, diarrhea, blood/plasma loss (trauma and surgery) and polyuria. For fever, formulas are available to estimate insensible losses, which approach 10 ml/kg per 1°C rise in temperature above 37°C [7]. Careful analysis of fluid intake and output, as well as estimations of insensible losses, may then be necessary to correct derangements in fluid status of the patient. This assessment may also include obtaining a body weight on a daily basis, although the latter is confounded by food intake. In the intensive care unit (ICU), obtaining daily weight can be difficult and, although clinically relevant in several (renal) patient groups, can be poorly associated with fluid balances [7].

Assessing hypovolemia is hard in hospitalized patients and many methods, from simple physical to complex physiological, have been described. Hypovolemia is often, rightfully or erroneously, considered in case of hypotension, low filling pressures, low central or mixed venous hemoglobin oxygen saturation, or need of isotropic or vasopressor drugs, which are clinical triggers for fluid infusion, even though this may benefit the patient only in the presence of preload and fluidresponsiveness, irrespective of circulating plasma volume. Fluid responsiveness is defined by an increase in stroke volume or cardiac output by a fluid challenge or, preferably, passive leg raising to increase venous return. Incorporation of this strategy into decision making on fluid loading, even in a patient in shock, may help to prevent potentially harmful overhydration.

Although not beyond doubt, severe hypovolemia may include the interstitial and eventually even the intracellular space, and repletion of these volumes can be accomplished by intravenous fluid administration [8,9]. Hypovolemia and decreased organ perfusion leading to tissue hypoxia may lead to shock. Fluid losses otherwise always include plasma water constituents, including sodium, potassium and magnesium, and their anions, such as chloride and bicarbonate, and other electrolytes. Electrolyte losses thus accompany fluid losses, for instance perioperatively [8]. In addition, the postoperative state is characterized by non-osmotic release of vasopressin, and activation of the renin-angiotensin-aldosterone system and the hypothalamic-pituitary-adrenal axis, with release of hormones which promote renal retention of water and sodium, to compensate in part for extrarenal losses.

Repletion of lost plasma should ultimately include all components, to prevent electrolyte and acid–base derangements, even independently of hypovolemia- and shock-induced alterations in production and elimination of acids and shifts in electrolyte distributions between intra- and extravascular spaces. One should take into account the electrolyte status of a patient before choosing an optimal fluid type for resuscitation and maintaining euvolemia.

### Treating hypovolemia and shock

In this section, we will discuss fluid doses and types from a ‘crystalloid’ perspective. Crystalloid fluid loading may decrease mortality in hypovolemic patients, for instance after hemorrhage [10], but the optimal type and dose of crystalloid fluids remains to be defined in a variety of clinical settings.

In contrast to crystalloids, colloids remain in the circulation and increase intravascular volume by raising colloid osmotic pressure. They therefore have a three- to fourfold greater (sustained) plasma volume expanding and hemodynamic effects compared to crystalloids, for a given infusion volume [5,11-16]. A ratio of 1:3 to 1:4 for colloids versus crystalloids to result in similar plasma volume expansion may even hold true in conditions such as sepsis where increased permeability may limit intravascular retention of colloid substances, provided that similar hemodynamic endpoints associated with plasma volume expansion are reached [11,13,17-20]. Authors have suggested, however, that in daily practice only 10 to 40% more crystalloids than colloids are infused, and results in similar resuscitation goals and outcomes [4,6,21-26]. Hence, the three to fourfold increased requirements for crystalloids to replace a given plasma volume are probably somewhat overestimated in clinical practice and are likely to be less. In hemorrhaged volunteers, 100 to 200% of Ringer’s acetate may suffice to replace lost blood [27]. However, in other studies only 20% of infusion volumes of crystalloids are retained intravascularly and more than 60% equilibrates with the extravascular space within minutes or is excreted by the kidney within hours after administration [13,15,28]. Albumin only saves about 40% of saline volume loading in the so-called SAFE study (a large Australian study to investigate safety of albumin solution in intensive care), together with a slightly higher central venous and arterial blood pressure, and lower heart rate [21].

Indeed, it is likely that the simpler the hemodynamic targets are, like static hydrostatic pressures, the less they reflect plasma volume, and the less strictly they are used to guide resuscitation, the lower is the anticipated volume difference between resuscitation fluid types [25]. This largely explains some of the seeming discrepancies noted above and, conversely, implies that the hemodynamic effects of crystalloids are much less than of roughly equal amounts of infused colloids. Otherwise, the greater the hypovolemia, the slower the clearance and longer the half life are of a given infused crystalloid fluid volume, as calculated from kinetics of hemoglobin concentrations [29]. Finally, accommodation by unstressed volume in compliant veins attenuates the plasma volume expanding effect of infusion fluids.

Although fluid therapy is essential in the treatment of shock and tissue hypoperfusion, overhydration and grossly positive fluid balances are, generally, considered harmful by contributing to organ dysfunction after sepsis, trauma or surgery, raising the question which fluid type and dose can optimize hemodynamics and volume status, with the lowest risk of fluid overloading [30-34]. Crystalloids do not seem inferior to colloids, that is increase mortality, in surgical and critically ill patients with hypovolemia or shock of various origins [5,11-16]. Some of excessively infused crystalloid fluids may be excreted by the kidneys, sometimes even more than colloid fluids, and thereby contribute to limit an increasing fluid balance over hours and days [15]. It is, however, also generally suggested that resuscitation with crystalloids results in a more positive fluid balance and risk for (pulmonary) edema formation than that with colloids, even though the magnitude of this effect has probably also been overemphasized in the past [17,24-26,30].

The propensity of crystalloid fluids to increase pulmonary edema formation, even in face of increased pulmonary vascular permeability and edema (in sepsis) at baseline, has not been confirmed, provided that fluid loading was done in the steep (rather than the plateau) portion of cardiac and pulmonary vascular filling [35]. The tendency for pulmonary edema formation, as inferred from radiologic and gas exchange changes in other studies, can thus be explained in part by fluid loading in the plateau of cardiac filling (fluid unresponsiveness) and pulmonary vascular filling (pulmonary congestion), independent of fluid type [17,35]. During hypothermia after cardiac arrest, more isotonic crystalloids were needed as compared to starch and hypertonic saline, but this did not lead to cerebral edema [36]. Nevertheless, it cannot be denied that overzealous crystalloid fluid loading has somewhat greater tendency for development of peripheral edema (and compartment syndromes) than resuscitation with colloids, against similar hemodynamic targets, particularly after multiple trauma, and that this can be detrimental [16,34,37,38]. Table 3 summarizes the pros and cons of clinical use of (balanced or unbalanced) crystalloid fluids, related to fluid volumes.

**Table 3:** Pros and cons of (unbalanced) crystalloid fluids leading to under- or over-resuscitation in clinical practice

**Too little**	**Too much**
Tissue hypoxygenation	Tissue edema and hypoxygenation
Risk for acute kidney injury	Compartment syndromes and renal dysfunction
Lactate and unmeasured anion acidosis	Hyperchloremic metabolic acidosis and risk forhypernatremia
Gastrointestinal disturbances	Anastomotic leakage; diarrhea and other gastrointestinal disturbances
	Pulmonary edema; hepatic congestion and injury
	Prolonged mechanical ventilation

Compared to isotonic crystalloids, relatively small volumes of hypertonigc solutions may suffice in restoring euvolemia and hemodynamics without grossly expanding the interstitial space, in hypovolemia and shock [9,13,30]. Relatively small-volume hypertonic saline 3 to 7% (whether or not accompanied by colloid fluids) is commonly used in emergency medicine for patients with traumatic hemorrhage and/or severe traumatic brain injury [39-42]. Hypertonic solutions may require less volumes than isotonic crystalloids in maintaining hemodynamics, also perioperatively, but may not carry other advantages in that setting [43]. This type of solution exerts a rapid volume expanding effect, which is larger than that of isotonic solutions, and a positive inotropic effect without too much dilution of coagulation factors, and perhaps prevents, delays or ameliorates a detrimental increase in intracranial pressure [40]. By limiting volume needs, it may also prevent development of abdominal compartment syndrome, as compared to Ringer’s lactate during resuscitation from burn injury [37]. The hypertonic solutions may also have anti-inflammatory properties [44,45]. Nevertheless, the formal large-scale clinical studies on the subject are inconclusive, as their use may not decrease morbidity or mortality [39-43].

Hypertonic lactate solutions are also available and, like other hypertonic solutions, may have positive inotropic effects on the heart [37,46]. Hypertonic saline 3 to 7% can also be regarded as osmotherapy when applied for reducing intracranial pressure, as a primary drug or when mannitol has failed, which is the more common application [40,47,48]. However, serum sodium levels above 160 mmol/lare unlikely to further decrease cerebral edema [47]. The treatment may be even more effective in controlling intracranial hypertension than mannitol, but may not improve outcomes [40,48]. Obviously, the major adverse effect is severe hypernatremia, which is sometimes hard to control. Conversely, hypotonic fluids should be avoided in the condition, carrying the risk of edema aggravation, whereas under-resuscitation and resultant hypotension are also associated with a worse outcome. Management thus requires a careful balance, helped by hemodynamic and intracranial pressure monitoring, between systemic hemodynamics and cerebral perfusion pressure.

### Perioperative fluid therapy strategies: dose and targets

Dosing and guiding emergency or perioperative crystalloid fluid therapy is still surrounded by controversy. In spite of the less plasma volume expanding and hemodynamic effects of crystalloids versus colloids, for a given fluid infusion volume, a restricted (usually defined as <7 ml/kg/h) fluid policy has been suggested to result in less complications than a standard or more liberal fluid strategy, regardless of the principal type of solution used, in randomized studies in surgical patients [30,33,49-55]. The surgery may involve abdominal, pancreatic, colorectal, gall bladder, vascular, and hip and knee surgery, whereas definitions of policies for fluid amounts differ across studies [49-57]. The complications prevented may include anastomotic leaks, gastrointestinal disturbances with nausea and vomiting, infections, pulmonary complications, and others [53,56,58]. Indeed, many of these complications may result from overhydration, particularly by crystalloids, whereas fluid restriction often refers to limitation of crystalloids [22,30,49,57,59].

However, too restrictive fluid therapy may result in under-resuscitation and potentially associated adverse sequelae, again including gastrointestinal hypoperfusion, anastomotic leaks, nausea, vomiting, and others, which can be prevented in part by more liberal (colloid or crystalloid) fluid loading [30,54,59-63]. Perioperative, goal-directed hemodynamic optimization by (mostly colloid, sometimes crystalloid) fluids may better reduce postoperative complications, because of dosing fluids to individual needs in the patient and by taking individual cardiac reserve into account, than either liberal or restrictive policies [2,4,22,52,53,61,64-66].

Hence, the debate on fluid volumes and hemodynamic targets is confounded by the type of fluid, with increasing doubts on the safety of synthetic colloids, at least in sepsis and perhaps not in trauma and surgery, thereby favoring crystalloids [25,67]. Also, the risk to benefit ratio of a fluid type may be determined by the severity of hypovolemia and shock during which it is infused. In any case, preoperative starvation commonly includes fluids and may contribute to postoperative hypovolemia [68], and authors have studied the effect of preoperative optimization with help of Ringer’s lactate and found that this policy reduced postoperative morbidity [69]. In the emergency treatment of trauma, avoidance of overzealous crystalloid fluid therapy may also be associated with better outcomes [34].

Concluding this section, one should give crystalloid fluid challenges as a first choice on clinical grounds, for resuscitation from hypovolemia or shock, by utilizing rapid infusions of 500 to 1000 ml, provided that the patient is likely to be fluid responsive at baseline or indeed responds to fluids during loading. In the maintenance phase, measurable losses augmented by 1 l per day for insensible losses mostly result in infusion of about 2 to 2.5 l per day. For resuscitation and maintenance, special considerations obviously apply to brain injury, trauma and surgery, when restriction of fluids and electrolytes may be preferred to limit a positive fluid balance, and to cardiac failure and edema-forming states, when a negative fluid balance rather than maintaining the balance is aimed at.

### Composition and clinical use of various crystalloid fluids

We will now discuss varying crystalloid fluid types and their properties. Normal or physiological saline was first described and applied in 1831, and since then 0.9% sodium chloride (NaCl) is the most widely used intravenous fluid in the hospital, in spite of doubts on its ‘normal’ or ‘physiological’ properties [70]. Adding lactate as a buffer to better resemble electrolyte composition of human plasma yields Ringer’s lactated (or Hartmann’s) solution, yet is still slightly hypotonic with the ability to lower serum osmolality [71]. Hypotonic solutions, including colloids (albumin) and crystalloids, may increase (the risk for) cerebral edema in traumatic brain injury, which seems determined in part by osmotic pressure, unless rapid excretion of hypotonic urine maintains plasma osmolality [71,72]. Infusion of hypotonic dextrose 5% in water is thus also contraindicated in traumatic brain injury, if decreasing plasma osmolality [11]. The solution has a minimum effect on plasma volume and is therefore not a suitable resuscitation fluid either. Therefore, normal saline or isotonic balanced solutions should be preferred to treat hypovolemia in brain injury. Sodium bicarbonate is available in hypertonic solutions. Even though the benefit of treating metabolic (lactic) acidosis during ischemia and resuscitation is controversial, sodium bicarbonate is still widely used to ameliorate metabolic acidosis from various other origins and adjunctive treatment to keep the pH above 7.15 to 7.20 in high risk patients remains commonly recommended. Sodium bicarbonate is also used in the prevention of contrast nephropathy, although probably hardly more effective than prehydration with normal saline [73-75]. Conversely, infusion may not be harmless, and result in hypernatremia and overshoot alkalosis if (too much of) hypertonic solutions are used.

Commonly applied crystalloid solutions of osmolality, cationic and anionic composition are described in Table 4.

**Table 4:** Commonly applied crystalloid solutions: osmolality, cationic and anionic composition

	**Osmolality (mOsm/kg)**	**pH**	**Na**^**+**^**(mmol/l)**	**K**^**+**^**(mmol/l)**	**Ca**^**2+**^**(mmol/l)**	**Mg**^**2+**^**(mmol/l)**	**HCO**_**3 **_**(mmol/l)**	**Lactate (mmol/l)**	**Other buffer (mmol/l)**	**Cl**^**-**^**(mmol/l)**
0.9% saline	308	5.5	154							154
Ringer’s	309		147	4	5					156
Ringer’s lactate	273	6.5	130	5.4	2.7			29		109
Ringer’s lactate	273	6.5	130	5.4	2.7				29^a^	
Hartmann’s	280	6.5	131	5.4	2			29		112
Modified	290		145	4	2.5	1			24^a^_1_	127
Balanced	294	5.5	140	5		1.5			50^b^	98
Fluids	299	5.5	140	10	5	3		8	47^a^	103
1.4% Na bicarbonate	333	>7.0	167				167			
4.2% Na bicarbonate	1,000	>7.0	500				500			
8.4% Na bicarbonate	2,000	>7.0	1,000				1,000			
3% saline	1,026		513							513
7% saline	2,394		1,197							1,197

^a^acetate, ^a^_1_ with 5 mmol/l maleate, ^b^27 mmol/l as acetate and 23 mmol/l as gluconate.

#### Chloride and balanced solutions

The chloride content of normal saline (0.9% NaCl), a so-called unbalanced solution, that is 154 mmol/l, is much higher than found in plasma (101 to 110 mmol/l) and higher than the so-called balanced solutions as (modified) Ringer’s or Hartmann’s, where some of the anions are buffering lactate, acetate, or gluconate instead of chloride [76]. The strong ion difference of these latter solutions is thus higher than that of saline (for example closer to that of plasma, 42 mEq/l). Even though the pH of these solutions may not differ much, their *in vivo* effects on acid–base balance are profoundly different [77]. Indeed, saline infusion commonly leads to hyperchloremic metabolic acidosis and contributing to hyperkalemia, particularly when large volumes are infused, whereas infusion of balanced solutions does not carry this effect [76,78-86].

Although the use of saline may result in organ dysfunction (shown extensively in animal studies) and may contribute to gastrointestinal symptoms after surgery, the true detriments of hyperchloremic acidosis in clinical studies remain uncertain [76]. Furthermore, plasma dilution, by any type of infusion solution not containing bicarbonate (or precursor buffer anions), including dextrose 5% in water, decreases strong ion differences and thus blood pH [87].

However, Shaw *et al*., in a large retrospective study, using propensity-matched samples, recently found that use of normal saline resulted in a higher mortality than using balanced solutions during and following surgery (5.6% versus 2.9%), preventing hyperchloremic metabolic acidosis as well [88]. In addition, the administration of balanced solutions was associated with less morbidity as shown by fewer days on the ventilator, less need for fluid, less need for transfusions, fewer major infections and less need for renal replacement therapy. This suggests that balanced solutions are favored to saline considering the risk of metabolic derangements as hyperchloremic metabolic acidosis and patient morbidity, and clinical course. However, the mechanisms of some of these effects in humans remain unclear and further research on effect on morbidity and mortality is needed [76,89].

Finally, we caution against the use of potassium-containing balanced solutions in anuric patients with renal insufficiency since the infused potassium cannot be excreted ultimately.

#### Lactate is reshuttled via the Cori cycle

Hepatic uptake results in gluconeogenesis, an energy costing process. Oxidation is the fate of 30 to 60% of infused lactate, even in septic and cardiogenic shock, thereby sparing other substrates [90]. Lactate may thus serve as a fuel for liver, and even heart and brain, particularly when other substrates are less available [46]. Infusion of (large quantities of) lactate-containing infusions may transiently and slightly elevate the serum lactate level (but do not decrease pH), particularly in case of liver dysfunction [46,91,92]. It may increase glucose levels in patients with poorly controlled diabetes mellitus or otherwise enhanced gluconeogenesis. Use of Ringer’s lactate is safer and saves more lives than use of starch solutions, when compared in the resuscitation of septic shock [67].

Ringer’s acetate solution closely resembles Ringer’s lactate. Acetate, a component of Ringer’s acetate and other solutions, is, similarly to lactate, not metabolically inert. It enters the tricarboxylic acid cycle and is combusted, thereby consuming oxygen by partly replacing other substrates. Indeed, when infused, about 90% is oxidized. Acetate as a buffer, so that, upon infusion, the bicarbonate concentration and pH rises, similarly as when equimolar bicarbonate is infused, in acidosis following diarrhea or renal insufficiency [93]. Also, acetate carries vasodilating and myocardial depressant properties, and may thus be harmful in patients with shock [94], although this has been denied in other studies [95,96]. In fact, it has recently been demonstrated that resuscitation from septic shock with Ringer’s acetate results in greater survival than resuscitation with colloid, but this can be attributed to adverse effects of starches [25].

#### Renal effects

On the one hand, crystalloid solutions better preserve diuresis and renal function, and prevent need for renal replacement therapy than colloids in the critically ill [24], but not in healthy volunteers [15], probably by a fall in plasma colloid osmotic pressure promoting glomerular filtration. However, crystalloid fluid overloading *per se* may be associated with increased need for renal replacement therapy [16,76].

Recent evidence suggests that this may also depend on the type of crystalloid administered and the evidence for a renal protective effect of balanced (versus unbalanced) crystalloids is cumulating. Hypotonic Ringer’s lactate infusion in healthy volunteers is more rapidly excreted via urine than normal saline (requiring hours), but this can be attributed in part to hypotonicity of the fluid rather than prevention of hyperchloremia, even though normochloremia may protect against a fall in measured renal (and gastric) blood flow associated with hyperchloremia [76,81,83,97]. Animal experiments indeed suggest that hyperchloremia may increase renal vascular resistance and decrease blood flow following a direct renal vasoconstrictive effect by the chloride anion. Hadimioglu *et al*. compared in a randomized controlled trial balanced fluids with normal saline as intraoperative fluid replacement during renal transplant surgery, and showed that only saline decreased pH, bicarbonate and base excess [92]. This and other studies did not demonstrate a difference in the effects on renal function, but these studies may have been underpowered [84,92].

In contrast, a recent large sequential cohort study suggests that the use of a chloride-restricted fluid regimen of (modified) Ringer’s lactate solution (versus chloride liberal and saline-based regimens) did not decrease mortality, but decreased acute kidney injury and need for renal replacement therapy [98]. In the study by Shaw *et al*., mentioned above [88], administration of balanced solutions is associated with a reduction of need of renal replacement therapy after laparotomy from 4.8 to 1.0% (*P*<0.001), even after adjustment for confounders. The incidence of tubular injury and acute kidney injury in sodiumbicarbonate-treated cardiac surgery patients may be lower than when equimolar saline is used [99,100]. However, the implications for hospitalized patients with risk factors for renal dysfunction still need to be firmly established.

#### Immune depression and coagulation

Another controversy relates to alleged immune effects of balanced versus unbalanced solutions [76]. It has been suggested that normal, unbalanced saline has proinflammatory properties, relating to neutrophil activation [44,101]. This may contribute to detrimental effects in trauma and sepsis resuscitation. Ringer’s lactate may also be proinflammatory but it appeared that the D-lactate in racemic mixtures was largely responsible, so that its elimination abolished that effect [101,102]. Hypertonic saline has been claimed to have anti-inflammatory properties, but this is not beyond doubt either [101]. In contrast, the chloride anion has been suggested to impair coagulation [76,81]. However, saline and Ringer’s lactate (−based colloid solutions) equally induced, *ex vivo*, a hypercoagulable state at 20 to 40% dilution of blood and decreased coagulability at 60% dilution [103,104].

### Electrolyte solutions to treat electrolyte disorders

In hospitalized and surgical or critically ill patients, electrolyte disturbances are common [8,105]. Solutions are available to selectively supplement specific electrolyte deficits. Hypokalemic alkalosis is common and risk factors include use of diuretics, vomiting and diarrhea, and others, leading to chloride depletion. The condition may be accompanied by hypomagnesemia and may be a risk factor for cardiac depression, tissue hypoxygenation and rhythm disturbances. Correction of hypokalemia may be more successful by potassium supplementation when hypomagnesemia is simultaneously corrected. Potassium repletion can be accomplished by the oral and enteral route, if functioning, or otherwise by slow, intravenous administration of concentrated solutions at a maximum rate of 20 mEq/h, or higher if vitally indicated [106,107]. Infusion of potassium-containing balanced solutions may lead to dangerous hyperkalemia in the patient with renal insufficiency and anuria who cannot excrete the extra potassium load, although, on the other hand, prevention of acidosis may somewhat ameliorate a dangerous increase in serum potassium concentration [84,86,92,106].

Dysnatremia is often observed in the ICU setting and is associated with higher in-hospital mortality. Hypertonic saline is used in the urgent treatment, when indicated, of hyponatremia [108,109]. Glucose 5% is commonly used in the treatment of hypernatremia. For maintenance fluid therapy in the starving patient, the more complete and balanced solutions are more likely to maintain normal plasma electrolyte concentrations and acid–base balance than merely sodium-containing saline. To these fluids that resemble plasma but are not identical as far as their composition is concerned, glucose 5% can be added to meet some energy requirements too.

Hypocalcemia is also relatively frequent in postoperative and critically ill patients and may have adverse consequences [110]. It is likely that most critically ill patients are in a negative calcium balance, when immobilized, treated by citrate-based venovenous hemofiltration for acute kidney injury, and so on. A calcium deficit may lead to gradual bone loss. Hypocalcemia, even when adjusted for hypoalbuminemia, and resulting from a shift of extra- to intracellular calcium and hypovitaminosis D, may be associated with critical illness neuro- and myopathy [111]. Calcium is hard to include in (alkali) fluids, because it may easily become insoluble.

Continuous renal replacement therapy is a well known risk factor of hypophosphatemia in the critically ill. The latter may also be a feature of refeeding and hypophosphatemia is thus relatively frequently encountered in the critically ill patient (with and without renal failure) [111]. It may be associated with muscle weakness and prolonged need for mechanical ventilator support, and therefore requires prevention and correction with help of sodium-potassium-phosphate solutions, preferably via continuous infusions [112,113]. Currently, phosphate containing replacement fluids are available to prevent hypophosphatemia during continuous renal replacement therapy.

## Conclusion

Crystalloid fluids are the fluids of choice for expanding and maintaining plasma volume in hospitalized patients with hypovolemia or shock, although relatively large volumes have to be administered to increase circulating plasma volume and tissue oxygen delivery. Overzealous crystalloid administration increases the risk for fluid overload with hypernatremia, peripheral and pulmonary edema, and compartment syndromes. Hence, both under-resuscitation and overhydration should be avoided by careful monitoring. The evidence that balanced solutions are superior, particularly in helping to prevent acute kidney injury and when isotonic, to unbalanced ones is increasing. Hypotonic solutions are generally contraindicated in conditions with or at risk for cerebral edema. In the maintenance phase of non-oral fluid infusion, solutions which more resemble plasma water composition than normal saline are also to be recommended. We finally recommend serum sodium-guided treatment by hypertonic saline for mannitol-refractory cerebral edema and intracranial hypertension.

## Abbreviations

Ca2+: Calcium; HCO3: Bicarbonate; HP04: Phosphate; HSO4: Hydrogen sulfate; ICU: Intensive care unit; K+: Potassium; Mg2+: Magnesium; Na: Sodium; SAFEstudy: Saline versus albuminfluid evaluation study.

## Competing interests

ABJG and CI received an unrestricted research grant from B Braun Medical (Melsungen, German) for this study. CI has received research grant and speaker fees from Fresenius (Bad Homburg, Germany), Baxter HealthCare (Newbury, UK) and B Braun Medical.

## Authors’ contributions

AS and ABJG brought about initial set-up, literature research and writing of the manuscript. CI provided comments and recommendations, and improved the quality and clarity of the manuscript. ABJG was the supervising author. All authors read and approved the final manuscript.

## References

[B1] Lobo DN Dube MG Neal KR Simpson J Rowlands BJ Allison SP Problems with solutions: drowning in the brine of an inadequate knowledge base Clin Nutr 2001 2 125 130 10.1054/clnu.2000.0154 11327739

[B2] Grocott MPW Mythen MG Gan TJ Perioperative fluid management and clinical outcomes in adults Anesth Analg 2005 2 1093 1106 10.1213/01.ANE.0000148691.33690.AC 15781528

[B3] Finfer S Liu B Taylor C Bellomo R Billot L Cook D Du B McArthur C Myburgh J SAFE TRIPS Resuscitation fluid use in critically ill adults: an international cross-sectional study in 391 intensive care units Crit Care 2010 2 R185 10.1186/cc9293 20950434 PMC3219291

[B4] Morris C Rogerson D What is the optimal type of fluid to be used for peri-operative fluid optimization directed by oesophageal Doppler monitoring? Anaesthesia 2011 2 819 827 10.1111/j.1365-2044.2011.06775.x 21668911

[B5] Perel P Roberts I Colloids versus crystalloids for fluid resuscitation in critically ill patients Cochrane Database Syst Rev 2007 2 CD000567 10.1002/14651858.CD000567.pub6 23450531

[B6] Manz F Johner SA Wentz A Boeing H Remer T Water balance throughout the adult life span in a German population Br J Nutr 2012 2 1673 1681 10.1017/S0007114511004776 21920064

[B7] Schneider AG Baldwin I Freitag E Glassford N Bellomo R Estimation of fluid status changes in critically ill patients: fluid balance chart or electronic bed weight J Crit Care 2012 2 745 22341728 10.1016/j.jcrc.2011.12.017

[B8] Polderman KH Girbes ARJ Severe electrolyte disorders following cardiac surgery: a prospective controlled observational study Crit Care 2004 2 R459 R466 10.1186/cc2973 15566592 PMC1065069

[B9] Brandstrup B Svensen C Engquist A Hemorrhage and operation cause a contraction of the extracellular space needing replacement – evidence and implications? A systematic review Surgery 2006 2 419 432 10.1016/j.surg.2005.07.035 16546507

[B10] Spoerke N Michalek J Schreiber M Brasel KJ Vercruysse G MacLeod J Dutton RP Duchesne JC McSwain NE Muskat P Johannigamn J Cryer HM Tillou A Cohen MJ Pittet JF Knudson P De Moya MA Tieu B Brundage S Napolitano LM Brunsvold M Sihler KC Peitzman AB Zenait MS Sperry J Alarcon L Croce MA Minei JP Stewart RM Trauma Outcomes Group Crystalloid resuscitation improves survival in trauma patients receiving low ratios of fresh frozen plasma to packed red blood cells J Trauma 2011 2 S380 S383 21814108 10.1097/TA.0b013e318227f1c5

[B11] Lamke L-O Liljedahl S-O Plasma volume changes after infusion of various plasma expanders Resuscitation 1976 2 93 102 10.1016/0300-9572(76)90029-0 69313

[B12] Jones SB Whitten CW Monk TG Influence of crystalloid and colloid replacement solutions on hemodynamic variables during acute normovolemic hemodilution J Clin Anesth 2004 2 11 17 10.1016/j.jclinane.2003.03.003 14984854

[B13] Hahn RG Volume kinetics for infusion fluids Anesthesiology 2010 2 470 481 10.1097/ALN.0b013e3181dcd88f 20613481

[B14] Gondos T Marjanek Z Ulakcsai Z Szabó Z Bogár L Károlyi M Gartner B Kiss K Havas A Futó J Short-term effectiveness of different volume replacement therapies in postoperative hypovolaemic patients Eur J Anaesthesiol 2010 2 794 800 10.1097/EJA.0b013e32833b3504 20520555

[B15] Lobo DN Stanga Z Aloysius MM Wicks C Nunes QM Ingram KL Risch L Allison SP Effect of volume loading with I liter intravenous infusions of 0.9% saline, 4% succinylated gelatin (Gelofusine) and 6% hydroxyethyl starch (Voluven) on blood volume and endocrine responses: a randomized, three-way crossover study in healthy volunteers Crit Care Med 2010 2 464 470 10.1097/CCM.0b013e3181bc80f1 19789444

[B16] James MFM Michell WL Joubert IA Nicol AJ Navsaria PH Gillespie RS Resuscitation with hydroxyethyl starch improves renal function and lactate clearance in penetrating trauma in a randomized controlled study: the FIRST trial (fluids in resuscitation of severe trauma) Br J Anaesth 2011 2 693 702 10.1093/bja/aer229 21857015

[B17] Rackow EC Falk JL Fein A Siegel JS Packman MI Haupt MT Kaufman BS Putnam D Fluid resuscitation in circulatory shock: a comparison of the cardiorespiratory effects of albumin, hetastarch, and saline solutions in patients with hypovolemic and septic shock Crit Care Med 1983 2 839 850 10.1097/00003246-198311000-00001 6194934

[B18] Ernest D Belzberg AS Dodek PM Distribution of normal saline and 5% albumin infusions in cardiac surgical patients Crit Care Med 2001 2 2299 230 10.1097/00003246-200112000-00011 11801830

[B19] Verheij J van Lingen A Beishuizen A Christiaans HM de Jong JR Girbes AR Wisselink W Rauwerda JA Huybregts MA Groeneveld AB Cardiac response is greater for colloid than saline fluid loading after cardiac or vascular surgery Intensive Care Med 2006 2 1030 1038 10.1007/s00134-006-0195-5 16791665

[B20] Trof RJ Sukul SP Twisk JW Girbes AR Groeneveld AB Greater cardiac response of colloid than saline fluid loading in septic and non-septic critically ill patients with clinical hypovolemia Crit Care Med 2010 2 697 701 10.1007/s00134-010-1776-x PMC2837190 20165941

[B21] Finfer S Bellomo R Boyce N French J Myburgh J Norton R SAFE Study Investigators A comparison of albumin and saline for fluid resuscitation in the intensive care unit N Engl J Med 2004 2 2247 2256 15163774 10.1056/NEJMoa040232

[B22] Senagore AJ Emery T Luchtefeld M Kim D Dujovny N Hoedema R Fluid management for laparoscopic colectomy: a prospective, randomized assessment of goal-directed administration of balanced salt solution on hetastarch coupled with an enhanced recovery program Dis Colon Rectum 2009 2 1935 1940 10.1007/DCR.0b013e3181b4c35e 19934912

[B23] Magder S Potter BJ De Varennes B Doucette S Fergusson D Canadian Critical Care Trials Group Fluids after cardiac surgery: a pilot study of the use of colloids versus crystalloids Crit Care Med 2010 2 2117 2124 10.1097/CCM.0b013e3181f3e08c 20802322

[B24] Bayer O Reinhart K Sakr Y Kabisch B Kohl M Riedemann NC Bauer M Settmacher U Hekmat K Hartog CS Renal effects of synthetic colloids and crystalloids in patients with severe sepsis: a prospective sequential comparison Crit Care Med 2011 2 1335 1342 10.1097/CCM.0b013e318212096a 21358396

[B25] Perner A Haase N Guttormsen AB Tenhunen J Klemenzson G Åneman A Madsen KR Møller MH Elkjær JM Poulsen LM Bendtsen A Winding R Steensen M Berezowicz P Søe-Jensen P Bestle M Strand K Wiis J White JO Thornberg KJ Quist L Nielsen J Andersen LH Holst LB Thormar K Kjældgaard AL Fabritius ML Mondrup F Pott FC Møller TP Hydroxyethyl starch 130/0.4 versus Ringer’s acetate in severe sepsis N Engl J Med 2012 2 124 134 10.1056/NEJMoa1204242 22738085

[B26] Bayer O Reinhart K Kohl M Kabisch B Marshall J Sakr Y Bauer M Hartog C Schwarzkopf D Riedemann N Effects of fluid resuscitation with synthetic colloids or crystalloids alone on shock reversal, fluid balance, and patient outcomes in patients with severe sepsis: a prospective sequential analysis Crit Care Med 2012 2 2543 2551 10.1097/CCM.0b013e318258fee7 22903091

[B27] Riddez L Hahn RG Brismar B Stranberg A Svensén C Hedenstierna G Central and regional hemodynamics during acute hypovolemia and volume substitution in volunteers Crit Care Med 1997 2 635 640 10.1097/00003246-199704000-00013 9142028

[B28] Jacob M Chappell D Hofmann-Kiefer K Helfen T Schuelke A Jacob B Burges A Conzen P Rehm M The intravascular volume effect of Ringer’s lactate is below 20%: a prospective study in humans Crit Care 2012 2 R86 10.1186/cc11344 22591647 PMC3580629

[B29] Zdolsek J Li Y Hahn RG Detection of dehydration by using volume kinetics Anesth Analg 2012 2 814 22 10.1213/ANE.0b013e318261f6ba 22763905

[B30] Doherty M Buggy DJ Intraoperative fluids: how much is too much? Br J Anaesth 2012 2 69 79 10.1093/bja/aes171 22661747

[B31] Holte K Sharrock NE Kehlet H Pathophysiology and clinical implications of perioperative fluid excess Br J Anaesth 2002 2 622 632 10.1093/bja/aef220 12393365

[B32] Sakr Y Vincent JL Reinhart K Groeneveld J Michalopoulos A Sprung CL Artigas A Ranieri VM Sepsis Occurence in Acutely Ill Patients Investigators High tidal volume and positive fluid balance are associated with worse outcome in acute lung injury Chest 2005 2 3098 3108 10.1378/chest.128.5.3098 16304249

[B33] Stewart RM Park PK Hunt JP McIntyre RC Jr McCarthy J Zarzabal LA Michalek JE National Institutes of Health/National Heart, Lung, and Blood Institute Acute Respiratory Distress Syndrome Clinical Trials Network Less is more: improved outcomes in surgical patients with conservative fluid administration and central venous pressure monitoring J Am Coll Surg 2009 2 725 735 10.1016/j.jamcollsurg.2009.01.026 19476825

[B34] Ley EJ Clond MA Srour MK Barnajian M Mirocha J Margulies DR Salim A Emergency department crystalloid resuscitation of 1.5 L or more is associated with increased mortality in elderly and nonelderly trauma patients J Trauma 2011 2 398 400 10.1097/TA.0b013e318208f99b 21307740

[B35] Aman J Groeneveld AB van Nieuw Amerongen GP Predictors of pulmonary edema formation during fluid loading in the critically ill with presumed hypovolemia Crit Care Med 2012 2 793 799 10.1097/CCM.0b013e318236f2df 22080639

[B36] Heradstveit BE Guttormsen AB Langørgen J Hammersborg SM Wentzel-Larsen T Fanebust R Larsson EM Heltne JK Capillary leakage in post-cardiac arrest survivors during therapeutic hypothermia - a prospective, randomised study Scand J Trauma Resusc Emerg Med 2010 2 29 10.1186/1757-7241-18-29 20500876 PMC2890528

[B37] Oda J Ueyama M Yamashita K Inoue T Noborio M Ode Y Aoki Y Sugimoto H Hypertonic lactated saline resuscitation reduces the risk of abdominal compartment syndrome in severely burned patients J Trauma 2006 2 64 71 10.1097/01.ta.0000199431.66938.99 16456437

[B38] Madigan MC Kemp CD Johnson JC Cotton BA Secondary abdominal compartment syndrome after severe extremity injury: are early, aggressive fluid resuscitation strategies to blame? J Trauma 2008 2 280 285 10.1097/TA.0b013e3181622bb6 18301187

[B39] Cooper DJ Myles PS McDermott FT Murray LJ Laidlaw J Cooper G Tremayne AB Bernard SS Ponsford J HTS Study Investigators Prehospital hypertonic saline resuscitation of patients with hypotension and severe traumatic brain injury: a randomized controlled trial JAMA 2004 2 1350 1357 10.1001/jama.291.11.1350 15026402

[B40] Strandvik GF Hypertonic saline in critical care: a review of the literature and guidelines for use in hypotensive states and raised intracranial pressure Anaesthesia 2009 2 9901003 10.1111/j.1365-2044.2009.05986.x 19686485

[B41] Bulger EM May S Brasel KJ Schreiber M Kerby JD Tisherman SA Newgard C Slutsky A Coimbra R Emerson S Minei JP Bardarson B Kudenchuk P Baker A Christenson J Idris A Davis D Fabian TC Aufderheide TP Callaway C Williams C Banek J Vaillancourt C van Heest R Sopko G Hata JS Hoyt DB ROC Investigators Out-of-hospital hypertonic resuscitation following severe traumatic brain injury JAMA 2010 2 1455 1464 10.1001/jama.2010.1405 20924011 PMC3015143

[B42] Bulger EM May S Kerby JD Emerson S Stiell IG Schreiber MA Brasel KJ Tisherman SA Coimbra R Rizoli S Minei JP Hata JS Sopko G Evans DC Hoyt DB ROC investigators Out-of-hospital hypertonic resuscitation after traumatic hemorrhagic shock. A randomized, placebo controlled trial Ann Surg 2011 2 431 441 10.1097/SLA.0b013e3181fcdb22 21178763 PMC3232054

[B43] McAllister V Burns KEA Znajda T Church B Hypertonic saline for peri-operative fluid management (review) Cochrane Data base Syst Rev 2010 2 CD005576 10.1002/14651858.CD005576.pub2 20091580

[B44] Rhee P Wang D Ruff P Austin B DeBraux S Wolcott K Burris D Ling G Sun L Human neutrophil activation and increased adhesion by various resuscitation fluids Crit Care Med 2000 2 74 78 10.1097/00003246-200001000-00012 10667502

[B45] Junger WG RHind SG Rizoli SB Cuschieri J Shiu MY Baker AJ Li L Shek PN Hoyt DB Bulger EM Resuscitation of traumatic hemorrhagic shock patients with hypertonic saline - without dextran - inhibits neutrophil and endothelial activation Shock 2012 2 341 350 10.1097/SHK.0b013e3182635aca 22777113 PMC3455119

[B46] Leverve XM Boon C Hakim T Anwar M Siregar E Mustafa I Half-molar sodium-lactate solution has a beneficial effect in patients after coronary artery bypass grafting Intensive Care Med 2008 2 1796 1803 10.1007/s00134-008-1165-x 18563389

[B47] Popper AH Hyperosmolar therapy for raised intracranial pressure N Engl J Med 2012 2 746 752 10.1056/NEJMct1206321 22913684

[B48] Kamel H Navi BB Nakagawa K Hemphill C Ko NU Hypertonic saline versus mannitol for the treatment of elevated intracranial pressure: a meta-analysis of randomized clinical trials Crit Care Med 2011 2 554 559 10.1097/CCM.0b013e318206b9be 21242790

[B49] Nisanevich V Felsenstein I ALmogy G Weissman C Einav S Matot I Effect of intraoperative fluid management on outcome after intraabdominal surgery Anesthesiology 2005 2 25 32 10.1097/00000542-200507000-00008 15983453

[B50] de Aguilar-Nasciamento JE Diniz BN Do Carmo AV Silveira EAO Silva RM Clinical benefits after the implementation of a protocol of restricted perioperative intravenous crystalloid fluids in major abdominal operations World J Surg 2009 2 925 930 10.1007/s00268-009-9944-2 19234737

[B51] González-Fajardo JA Mengibar L Brizuela JA Castrodeza J Vaquero-Puerta C Effect of postoperative restrictive fluid therapy in the recovery of patients with abdominal vascular surgery Eur J Vasc Endovasc Surg 2009 2 538 543 10.1016/j.ejvs.2009.01.010 19231249

[B52] WenKui Y Ning L JianFeng G WeiQin L ShaoQiu T Zhihui T Tao G JuanJuan Z FengChan X Hui S WeiMing Z Jie-Shou L Restricted peri-operative fluid administration adjusted by serum lactate level improved outcome after major elective surgery for gastrointestinal malignancy Surgery 2010 2 542 552 10.1016/j.surg.2009.10.036 20004445

[B53] Lobo SM Ronchi LS Oliveira NE Brandăo PG Froes A Cunrath GS Nishiyama KG Netinho JG Lobo FR Restrictive strategy of intraoperative fluid maintenance during optimization of oxygen delivery decreases major complications after high-risk surgery Crit Care 2011 2 R226 10.1186/cc10466 21943111 PMC3334772

[B54] Melis M Marcon F Masi A Sarpel U Miller G Moore H Cohen S Berman R Pachter HL Newman E Effect of intra-operative fluid volume on peri-operative outcomes after pancreaticoduodenectomy for pancreatic adenocarcinoma J Surg Oncol 2012 2 81 84 10.1002/jso.22048 21792977

[B55] Abraham-Nordling M Hjern F Pollack J Prytz M Borg T Kressner U Randomized clinical trial of fluid resuscitation in colorectal surgery Br J Surg 2012 2 186 191 10.1002/bjs.7702 21948211

[B56] Lobo DN Bostock KA Neal KR Perkins AC Rowlands BJ Allison SP Effect of salt and water balance on recovery of gastrointestinal function after elective colonic resection: a randomised controlled trial Lancet 2002 2 1812 1818 10.1016/S0140-6736(02)08711-1 12044376

[B57] Joshi GP Intraoperative fluid restriction improves outcome of major elective gastrointestinal surgery Anesth Analg 2005 2 601 605 10.1213/01.ANE.0000159171.26521.31 16037184

[B58] Schnüriger B Inaba K Wu T Eberle BM Belzberg H Demetriades D Crystalloids after primary colon resection and anastomosis at initial trauma laparotomy: excessive volumes are associated with anastomotic leakage J Trauma 2011 2 603 610 10.1097/TA.0b013e3182092abb 21610349

[B59] Varadhan KK Lobo DN Symposium 3: death by drowning. A meta-analysis of randomized controlled trials of intravenous fluid therapy in major elective open abdominal surgery: getting the balance right Proc Nutr Soc 2010 2 488 498 10.1017/S0029665110001734 20515521

[B60] Moretti EW Robertson KM El-Moalem H Gan TJ Intraoperative colloid administration reduces postoperative nausea and vomiting and improves postoperative outcomes compared with crystalloid administration Anesth Analg 2003 2 611 617 12538221 10.1097/00000539-200302000-00056

[B61] Bundgaard-Nielsen M Secher NH Kehlet H ‘Liberal” vs. ‘restrictive’ perioperative fluid therapy – a critical assessment of the evidence Acta Anaesthesiol Scand 2009 2 843 851 10.1111/j.1399-6576.2009.02029.x 19519723

[B62] Futier E Constantin J-M Petit A Chanques G Kwiatkowski F Flamein R Slim K Sapin V Jaber S Bazin JE Conservative vs restrictive individualized goal-directed fluid replacement strategy in major abdominal surgery. A prospective trial Arch Surg 2010 2 1193 1200 10.1001/archsurg.2010.275 21173294

[B63] Sharma CS Gupta V Dixi MB Sadhu S Jashi N Effect of perioperative intravenous crystalloid infusion on postoperative nausea and vomiting after laparoscopic cholecycstectomy J Anaesth Clin Pharmacol 2010 2 383 386

[B64] Srinivasa S Taylor MHG Sammour T Kahokehr AA Hill AG Oesophageal Doppler-guided fluid administration in colorectal surgery: critical appraisal of published clinical trials Acta Anaesthesiol Scand 2011 2 4 13 10.1111/j.1399-6576.2010.02308.x 21126237

[B65] Challand C Struthers R Sneyd JR Erasmus PD Mellor N Hosie KB Minto G Randomized controlled trial of intraoperative goal-directed fluid therapy in aerobically fit and unfit patients having major colorectal surgery Br J Anaesth 2012 2 53 62 10.1093/bja/aer273 21873370

[B66] Corcoran T Rhodes JE Clarke S Myles PSS Ho KM Perioperative fluid management strategies in major surgery: a stratified meta-analysis Anesth Analg 2012 2 640 651 10.1213/ANE.0b013e318240d6eb 22253274

[B67] Brunkhorst FM Engel C Bloos F Meier-Hellmann A Ragaller M Weiler N Moerer O Gruendling M Oppert M Grond S Olthoff D Jaschinski U John S Rossaint R Welte T Schaefer M Kern P Kuhnt E Kiehntopf M Hartog C Natanson C Loeffler M Reinhart K German Competence Network Sepsis (SepNet) Intensive insulin therapy and pentastarch resuscitation in severe sepsis N Engl J Med 2008 2 125 139 10.1056/NEJMoa070716 18184958

[B68] Osugi T Tatara T Yada S Tashiro C Hydration status after overnight fast as measured by urine osmolality does not alter the magnitude of hypotension during general anesthesia in low risk patients Anesth Analg 2011 2 1307 1313 10.1213/ANE.0b013e3182114df4 21415435

[B69] Cuthbertson BH Campbell MK Stott SA Elders A Hernández R Boyers D Norrie J Kinsella J Brittenden J Cook J Rae D Cotton SC Alcorn D Addison J Grant A OCCUS Study Group A pragmatic multi-centre randomized controlled trial of fluid loading in high-risk surgical patients undergoing major elective surgery - the FOCCUS study Crit Care 2011 2 R296 10.1186/cc10592 22177541 PMC3388651

[B70] Awad S Allison SP Lobo DN The history of 0.9% saline Clin Nutr 2008 2 179 188 10.1016/j.clnu.2008.01.008 18313809

[B71] Williams EL Hildebrand KL McCormick SA Bedel MJ The effect of intravenous lactated Ringer’s solution versus 0.9% sodium chloride solution on serum osmolality in human volunteers Anesth Analg 1999 2 999 1003 10320158 10.1097/00000539-199905000-00006

[B72] Hahn RG Drobin D Rapid water and slow sodium excretion of acetated Ringer’s solution dehydrates cells Anesth Analg 2003 2 1590 1594 14633525 10.1213/01.ANE.0000090543.37260.34

[B73] Hoste EAJ De Waele JJ Gevaert SA Uchino S Kellum JA Sodium bicarbonate for prevention of contrast-induced acute kidney injury: a systematic review and meta-analysis Nephrol Dial Transplant 2010 2 747 758 10.1093/ndt/gfp389 19703838

[B74] Klima T Christ A Marana I Kalbermatter S Uthoff H Burri E Hartwiger S Schindler C Breidthardt T Marenzi G Mueller C Sodium chloride vs. sodium bicarbonate for the prevention of contrast medium-induced nephropathy: a randomized controlled trial Eur Heart J 2012 2 2071 2079 10.1093/eurheartj/ehr501 22267245

[B75] Hewitt J Uniacke M Hansi NK Venkat-Raman G McCarthy K Sodium bicarbonate supplements for treating acute kidney injury Cochrane Database Syst Rev 2012 2 CD009204 10.1002/14651858.CD009204.pub2 PMC6481784 22696382

[B76] Guidet B Soni N Della Rocca G Kozek S Vallet B Annane D James M A balanced view of balanced solutions Crit Care 2010 2 325 10.1186/cc9230 21067552 PMC3219243

[B77] Omron EM Omron RM A physicochemical model of crystalloid infusion on acid–base status J Intensive Care Med 2010 2 271 280 10.1177/0885066610371633 20622258

[B78] McFarlane C Lee A A comparison of Plasmalyte 148 and 0.9% saline for intra-operative fluid replacement Anaesthesia 1994 2 779 781 7978133 10.1111/j.1365-2044.1994.tb04450.x

[B79] Scheingraber S Rehm M Sehmisch C Finsterer U Rapid saline infusion produces hyperchloremic acidosis in patients undergoing gynecologic surgery Anesthesiol 1999 2 1265 1270 10.1097/00000542-199905000-00007 10319771

[B80] Ho AM-H Karmakar MK Contardi LAH Ng SSW Hewson JR Excessive use of normal saline in managing traumatized patients in shock: a preventable contributor to acidosis J Trauma 2001 2 173 177 10.1097/00005373-200107000-00033 11468491

[B81] Wilkes NJ Woolf R Mutch M Mallett SV Peachey T Stephens R Mythen MG The effects of balanced versus saline-based hetastarch and crystalloid solutions on acid–base and electrolyte status and gastric mucosal perfusion in elderly surgical patients Anesth Analg 2001 2 811 816 10.1097/00000539-200110000-00003 11574338

[B82] Waters JH Gottlieb A Schoenwald P Normal saline versus lactated Ringer’s solution for intraoperative fluid management in patients undergoing abdominal aortic aneurysm repair: an outcome study Anesth Analg 2001 2 817 822 10.1097/00000539-200110000-00004 11574339

[B83] Reid F Lobo DN Willimas RN Rowlands BJ Allison SP (Ab)normal saline and physiological Hartmann’s solution: a randomized double-blind crossover study Clin Sci 2003 2 17 24 10.1042/CS20020202 12519083

[B84] O’Malley CMN Frumento RJ Hardy MA Benvenisty AI Brentjens TE Mercer JS Bennett-Guerrero E A randomized, double-blind comparison of lactate Ringer’s solution and 0.9% NaCl during renal transplantation Anesth Analg 2005 2 1518 1524 10.1213/01.ANE.0000150939.28904.81 15845718

[B85] Bellomo R Morimatsu H French C Cole L Story D Uchino S Naka T SAFE Study Investigators The effects of saline or albumin resuscitation on acid–base status and serum electrolytes Crit Care Med 2006 2 2891 2897 16971855 10.1097/01.CCM.0000242159.32764.86

[B86] Chua H-R Venkatesh B Stachowski E Schneider AG Perkins K Ladanyi S Kruger P Bellomo R Plasma-Lyte 148 vs 0.9% saline for fluid resuscitation in diabetic ketoacidosis J Crit Care 2012 2 138 145 10.1016/j.jcrc.2012.01.007 22440386

[B87] Lang W Zander R Prediction of dilutional acidosis based on the revised classical dilution concept for bicarbonate J Appl Physiol 2005 2 62 71 15591303 10.1152/japplphysiol.00292.2004

[B88] Shaw AD Bagshaw SM Goldstein SL Scherer LA Duan M Schermer CR Kellum JA Major complications, mortality, and resource utilization after open abdominal surgery: 0.9% saline compared to Plasma-Lyte Ann Surg 2012 2 821 829 10.1097/SLA.0b013e31825074f5 22470070

[B89] Burdett E Dushianthan A Bennett Guerrero E Cro S Gan TJ Grocott MP James MF Mythen MG O'Malley CM Roche AM Rowan K Perioperative buffered versus non-buffered fluid administration for surgery in adults Cochrane Database Syst Rev 2012 2 CD004089 23235602 10.1002/14651858.CD004089.pub2

[B90] Revelly J-P Tappy L Martinez A Bollmann M Cayeux M-C Berger MM Chioléro RL Lactate and glucose metabolism in severe sepsis and cardiogenic shock Crit Care Med 2005 2 2235 2240 10.1097/01.CCM.0000181525.99295.8F 16215376

[B91] Didwania A Miller J Kassel D Jackson EV Jr Chernow B Effect of intravenous lactated Ringer's solution infusion on the circulating lactate concentration: part 3. Results of a prospective, randomized, double-blind, placebo-controlled trial Crit Care Med 1997 2 1851 1854 10.1097/00003246-199711000-00024 9366769

[B92] Hadimioglu N Saadawy I Saglam T Ertug Z Dinckan A The effect of different crystalloid solutions on acid–base balance and early kidney function after kidney transplantation Anesth Analg 2008 2 264 269 10.1213/ane.0b013e3181732d64 18635497

[B93] Cash RA Toha KMM Nalin DR Huq Z Phillips RA Acetate in the correction of acidosis secondary to diarrhoea Lancet 1969 2 302 303 4184217 10.1016/s0140-6736(69)90060-9

[B94] Vincent JL Vanderweghem JL Degaute JP Berré J Dufaye P Kahn RJ Acetate-induced myocardial depression during hemodialysis for acute renal failure Kidney Int 1982 2 653 657 10.1038/ki.1982.225 7162037

[B95] Aoki K Yoshino A Yoh K Sekine K Yamazaki M Aikawa N A comparison of Ringer’s lactate and acetate solutions and resuscitative effects on splanchnic dysoxia in patients with extensive burns Burns 2010 2 1080 1085 10.1016/j.burns.2010.04.002 20483542

[B96] Davies PG Venkatesh B Morgan TJ Presneill JJ Kruger PS Thomas BJ Roberts MS Mundy J Plasma acetate, gluconate and interleukin-6 profiles during and after cardiopulmonary bypass: a comparison of Plasma-Lyte 148 with a bicarbonate-balanced solution Crit Care 2011 2 R21 10.1186/cc9966 21235742 PMC3222055

[B97] Chowdhury AH Cox EF Francis ST Lobo DN A randomized, controlled, double-blind crossover study on the effects of 2-L infusions of 0.9% saline and Plasma-Lyte®148 on renal blood flow velocity and renal cortical tissue perfusion in healthy volunteers Ann Surg 2012 2 18 24 10.1097/SLA.0b013e318256be72 22580944

[B98] Yunos NM Bellomo R Hegarty C Story D Ho L Bailey M Association between a chloride-liberal vs chloride-restrictive intravenous fluid administration strategy and kidney injury in critically ill adults JAMA 2012 2 1566 1572 10.1001/jama.2012.13356 23073953

[B99] Haase M Haase-Fielitz A Bellomo R Devarajan P Story D Matalanis G Reade MC Bagshaw SM Seevanayagam N Seevanayagam S Doolan L Buxton B Dragun D Sodium bicarbonate to prevent increases in serum creatinine after cardiac surgery: a pilot double-blind, randomized controlled trial Crit Care Med 2009 2 39 47 10.1097/CCM.0b013e318193216f 19112278

[B100] Neuhaus W Schick MA Bruno RR Schneiker B Förster CY Roewer N Wunder C The effects of colloid solutions on renal proximal tubular cells in vitro Anesth Analg 2012 2 371 374 10.1213/ANE.0b013e3182367a54 22025492

[B101] Khan R Kirschenbaum LA Larow C Astiz ME The effect of resuscitation fluids on neutrophil-endothelial cell interactions in septic shock Shock 2011 2 440 444 10.1097/SHK.0b013e3182336bda 21921833

[B102] Wu BU Hwang JQ Gardner TH Repas K Delee R Yu S Smith B Banks PA Conwell DL Lactated Ringer's solution reduces systemic inflammation compared with saline in patients with acute pancreatitis Clin Gastroenterol Hepatol 2011 2 710 717 10.1016/j.cgh.2011.04.026 21645639

[B103] Roche AM James MFM Bennett-Guerrero E Mythen MG A head-to-head comparison of the in vitro coagulation effects of saline-based and balanced electrolyte crystalloid and colloid intravenous fluids Anesth Analg 2006 2 1274 1279 99 10.1213/01.ane.0000197694.48429.94 16551936

[B104] Casutt M Kristoffy A Schuepfer G Spahn DR Konrad C **Effects on coagulation of balanced (130/0.4) and non-balanced (130/0.4) hydroxyethyl starch or gelatin compared with balanced Ringer’s solution: an **** *in vitro * ****study using two different viscoelastic coagulation tests ROTEM™ and SONOCLOT™.** Br J Anaesth 2010 2 273 281 10.1093/bja/aeq173 20659913

[B105] Lindner G Funk GC Hypernatremia in critically ill patients J Crit Car 2013 2 e11 e20 10.1016/j.jcrc.2012.10.037 22762930

[B106] Van De Vreede M Wilson SG Dooley MJ Intravenous potassium chloride use in hospitals: current practice J Pharm Pract Res Vol 2008 2 19 22

[B107] Philips DA Bauch TD Rapid correction of hypokalemia in a patient with an implantable cardioverter-defibrillator and recurrent ventricular tachycardia J Emerg Med 2010 2 308 316 10.1016/j.jemermed.2007.06.019 18375090

[B108] Adrogué HJ Madias NE The challenge of hyponatremia J Am Soc Nephrol 2012 2 1140 1148 10.1681/ASN.2012020128 22626822

[B109] Sakr Y Rother S Ferreira AM Ewald C Dünisch P Riedermann N Reinhart K Fluctuations in serum sodium levels are associated with an increased risk of death in surgical ICU patients Crit Care Med 2013 2 133 142 10.1097/CCM.0b013e318265f576 23128383

[B110] Kelly A Levine MA Hypocalcemia in the critically ill patient J Intensive Care Med 2012 2 166 177 10.1177/0885066611411543 21841146

[B111] Anastasopoulos D Kefaliakos A Michlopoulos A Is plasma calcium concentration implicated in the development of critical illness polyneuropathy and myopathy? Crit Care 2011 2 R247 10.1186/cc10505 22018206 PMC3334798

[B112] Charron T Bernard F Skrobik Y Simoneau N Gagnon N Leblanc M Intravenous phosphate in the intensive care unit: more aggressive repletion regimens for moderate and severe hypophosphatemia Intensive Care Med 2003 2 1273 1278 10.1007/s00134-003-1872-2 12845429

[B113] Geerse DA Bindels AJ Kuiper MA Roos AN Spronk PE Schultz MJ Treatment of hypophosphatemia in the intensive care unit: a review Crit Care 2010 2 R147 10.1186/cc9215 20682049 PMC2945130

