# Correction: Dose and type of crystalloid fluid therapy in adult hospitalized patients

**DOI:** 10.1186/2047-0525-3-3

**Published:** 2014-05-23

**Authors:** Annemieke Smorenberg, Can Ince, A B Johan Groeneveld

**Affiliations:** 1 Department of Intensive Care, Erasmus Medical Centre, ‘s Gravendijkwal 230, 3015 CE Rotterdam, The Netherlands

## 

After the publication of this work [1] it was brought to our attention that there was an error in Table two (Table 1 here) of the article, in which the table columns were misaligned. The correct version of the table is included here:

**Table 1:** Composition of bodyl fluids (in mmol/L)

	**Na**^**+**^	**K**^**+**^	**Ca**^**2+**^	**Mg**^**2+**^	**Cl**^**−**^	**HCO**_**3**_^**−**^	**HPO**_**4**_^**−**^	**HSO**_**4**_^**−**^	**Organic acid**	**Protein**
**Plasma**	142	4	5	2	101	27	2	1	6	16
**Plasma water**										
	153	4.3	5.4	2.2	109	29	2.2	1	6.5	17
**Interstitial water**										
	139	4	5	2	114	31	2	1	7	1
**Intracellular water**										
	10	160	2	26	3	10	100	20		65
